# Single-Centre Experience of Systemic Treatment with Vincristine, Ifosfamide, and Doxorubicin Alternating with Etoposide, Ifosfamide, and Cisplatin in Adult Patients with Ewing Sarcoma

**DOI:** 10.1155/2017/1781087

**Published:** 2017-12-28

**Authors:** Annelies Requilé, Paul M. Clement, Oliver E. Bechter, Herlinde Dumez, Annelies Verbiest, Raf Sciot, Daphne Hompes, Friedl Sinnaeve, Erik Van Limbergen, Patrick Schöffski

**Affiliations:** ^1^Department of General Medical Oncology, Leuven Cancer Institute, University Hospitals Leuven, Herestraat 49, 3000 Leuven, Belgium; ^2^Department of Pathology, University Hospitals Leuven, Leuven, Belgium; ^3^Department of Oncology, KU Leuven and University Hospitals Leuven, Leuven, Belgium; ^4^Department of Radiation Oncology, University Hospitals Leuven, Leuven, Belgium

## Abstract

The treatment of Ewing sarcoma (ES) in adult patients requires a multidisciplinary approach. Systemic therapy remains an important component of clinical management of this disease. ES is extremely rare in adult patients. Due to the rarity of the disease, no standard of care in terms of chemotherapy for the adult population exists, and the level of evidence for individual agents or some multidrug combinations is limited. Most regimens that are used in both adults and children include anthracyclines, etoposide, vincristine, cyclophosphamide, and ifosfamide. In this report, we describe our experience with the alternating use of triple combination therapies based on vincristine, ifosfamide, and doxorubicin (VIA) and an etoposide, ifosfamide, and cisplatin combination (VIP). We retrospectively evaluated the response rates, outcome, and tolerance of adult patients (*n* = 64) treated with VIA/VIP between 1990 and 2014. The patients included were treated with perioperative chemotherapy (53.1% neoadjuvant therapy and 17.2% adjuvant therapy) or had synchronous metastases at diagnosis (29.7%). Five-year overall survival rate was 52.2% for all patients, 72.2% for patients with localized disease, and 5.3% in patients with synchronous metastases. Overall response rate (ORR) was 37% after 2 cycles of VIA and 2 cycles of VIP. There were no patients with progressive disease (PD).

## 1. Introduction

The Ewing sarcoma family of tumors (ESFTs) formerly consisted of classical Ewing sarcoma (ES), Askin tumor, and primitive neuroectodermal tumor (PNET) [1] but is now referred to as Ewing sarcoma (ES). The term “PNET” is no longer used as a synonym for ES [2]. ES accounts for 5% of all childhood and adolescent cancers and is the second most common primary bone tumor (after osteosarcoma) in this age group. ES can also occur in soft tissues in about 15 to 30% of cases (extraosseous Ewing sarcoma, EES) [3]. In adult patients (>16 years), ES is very rare and is diagnosed in 8% of patients with a primary bone tumor [4].

ES is a chemotherapy-sensitive disease, as demonstrated by various trials in the last decades evaluating VACA (vincristine, dactinomycin, cyclophosphamide, and doxorubicin), VAC/IE (vincristine, doxorubicin, and cyclophosphamide alternating with ifosfamide-etoposide), VAIA (vincristine, dactinomycin, ifosfamide, and doxorubicin), EVAIA (adding etoposide to VAIA), and VIDE (omitting dactinomycin from EVAIA) [5–10].

The use of chemotherapy in combination with local treatment in patients with localized ES is associated with improved survival outcomes. Five-year overall survival (OS) in patients (all age groups) with nonmetastatic disease at initial presentation is ranging from 65 to 75% in published series [11]. The treatment of patients with primary metastatic disease, and of patients with ES failing initial multimodal treatment, is an unmet medical need with very unsatisfactory outcome. Of note, 25% of patients have metastatic spread at initial diagnosis [12]. The most common sites of metastases are the lungs (50%), bone (25%), and bone marrow (20%) [13].

Patient outcome has improved over the past decades because of more insight into combination chemotherapies, dose intensification, and better locoregional treatment modalities. The improvement in outcome is especially seen in younger patients (age < 15 years) [4]. Patients older than 16 years have a worse outcome, and in the adult patients, older age is a negative prognostic factor [14–16].

Several other prognostic factors are known. Disease extent (metastatic versus localized disease) is an important risk factor [17], as well as primary tumor site and size in case of localized disease. Axial bone localization is worse than peripheral localization [18, 19]. Higher tumor volume correlates with worse outcome [20].

**Figure 1:**
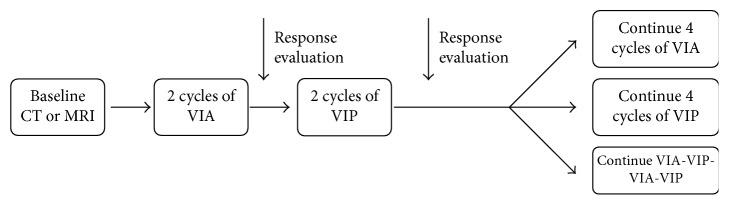
Outline of the chemotherapeutic regimen.

In our centre, adolescent and adult ES patients (age > 16 years) with localized and metastatic disease have been treated between 1990 and 2014 with two alternating chemotherapy regimens: VIA (vincristine, ifosfamide, and doxorubicin) and VIP (etoposide, ifosfamide, and cisplatin). After completing 2 cycles of VIA followed by an initial response assessment, the alternative protocol VIP is given for 2 further cycles, again followed by radiological assessment. The rationale for applying this alternating treatment scheme was to assess the sensitivity of the individual tumor to both the anthracycline- and the platinum-containing regimens. The treatment used after cycle 4 is based on the initial response assessment. If both regimens were found to be active, they were continued in an alternating schedule (Figure 1).

Between September 1990 and December 2014, a total of 64 patients were treated with this approach. We retrospectively evaluated the response rates to VIA/VIP in all these patients and report patient outcome in terms of overall survival. In patients receiving neoadjuvant treatment, pathological response rates were assessed in terms of percentage of necrosis. Adherence to the planned schedule and the total number of completed cycles and dose modifications are also reported.

## 2. Patients and Methods

### 2.1. Patient Selection

**Figure 2:**
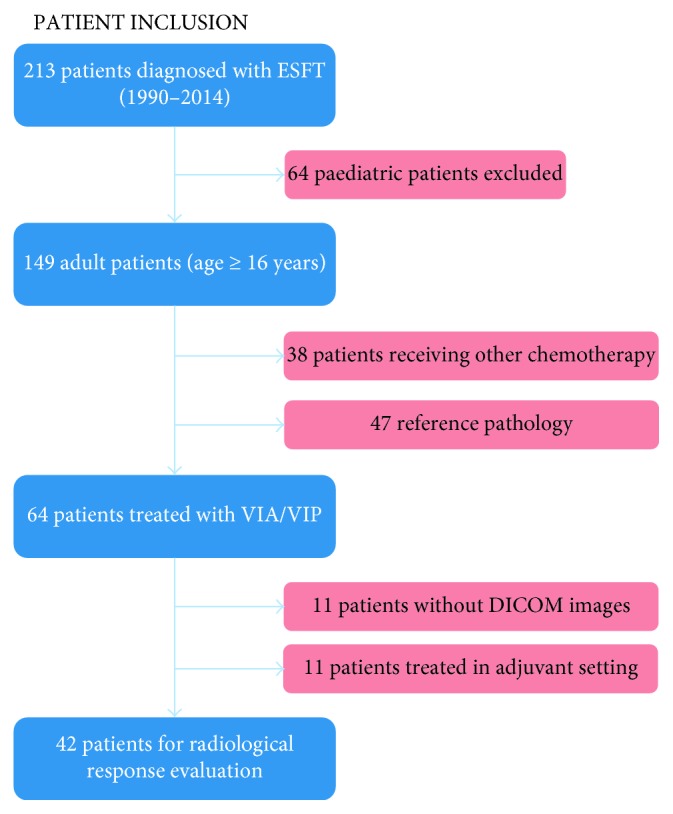
Inclusion of patients with diagnosis of ESFT treated in our hospital, aged 16 years and older, and treated with VIA/VIP. Further selection of radiological response evaluation was based on available DICOM images. ESFTs: Ewing's sarcoma family of Tumors; VIA: vincristine, ifosfamide, and doxorubicin; VIP: etoposide, ifosfamide, and cisplatin; DICOM: Digital Imaging and Communications in Medicine.

All patients diagnosed with ES received a reference number at our pathology department. We reviewed the medical records of all patients diagnosed between 1990 and 2014 and selected patients aged 16 years and older with diagnosis of ES who were treated with VIA/VIP (*n* = 64) (Figure 2). Patients with localized and metastatic disease were included. Patient demographics, disease localization, tumor characteristics, response to treatment, need for dose reduction or schedule modifications, and outcome in terms of response rate and survival were assessed.

We received approval of the ethics committee to perform this retrospective study.

### 2.2. Radiologic Evaluation

From patients with measurable disease according to the Response Evaluation Criteria in Solid Tumors (RECIST) version 1.1 (*n* = 42), all radiological images were reviewed, and disease evolution was assessed applying the very same criteria. All computed tomography (CT) or magnetic resonance imaging (MRI) scans were reviewed; target and nontarget lesion(s) were assigned and followed over time. Response evaluation was made after two initial cycles of VIA, after two cycles of VIP, and after completion of the chemotherapy. Bone lesions are per definition nonmeasurable according to RECIST, except when there is a measurable soft tissue component. We assessed radiological responses in bone-only disease, taking indirect signs of response into account such as remission of bone oedema on MRI, development of necrosis, and reduction of FDG-activity when PET-CT was performed.

### 2.3. Statistical Considerations

This is a monocentric, retrospective investigation. The main purpose of this evaluation was to compare the outcome and treatment adherence of our patient cohort with previously reported data in the literature, and results are given in a descriptive fashion.

Five-year overall survival (OS) for both metastatic and localized disease were assessed and displayed as Kaplan–Meier estimates. OS was defined as the interval between the date of histological diagnosis and the date of death from any cause or the date of the last follow-up.

### 2.4. Review of Literature

We searched http://www.pubmed.com for the MeSH terms “Ewing sarcoma,” “ESFT,” and adult patients.

## 3. Results

### 3.1. Patient Demographics

In the period of 1990 to 2014, 102 adult patients were diagnosed with ES at our hospital, and 64 of those received treatment with VIA/VIP (Figure 2). The main reason for choosing an alternative protocol in the other cases was comorbidity. Median age at diagnosis was 26 years (range of 16 to 67 years). In 51/64 patients (79.7%), the conventional pathological diagnosis was complemented by cytogenetic analysis looking for Ewing sarcoma (EWSR1) gene rearrangement by fluorescent in situ hybridization (FISH). FISH was found positive in tumor material originating from 45/64 patients (70.3%).

The main primary disease localizations were bone (central localization such as vertebral, sacral disease in 37.5% and peripheral localization in 21.9% of patients) and soft tissue (29.7%). Six out of 64 patients (9.4%) had an Askin tumor, defined as a primitive neuroectodermal tumor of the thoracopulmonary region [21]. A total of 29.7% of our cases had synchronous metastatic disease. Patient demographics are shown in Table 1.

### 3.2. Chemotherapy Application

All patients treated with the alternating VIA/VIP schedule started with 2 cycles of VIA (vincristine 1.2 mg/m^2^ on days 1, 8, and 15; ifosfamide 3000 mg/m^2^ on days 1–3; and doxorubicin 20 mg/m^2^ on days 1–3 in a three-weekly schedule). After interim response assessment, cycles 3 and 4 consisted of VIP (etoposide 100 mg/m^2^, ifosfamide 2000 mg/m^2^, and cisplatin 30 mg/m^2^ on days 1–3 also in a three-weekly cycle). The same dose was given to patients in the (neo)adjuvant or metastatic setting. To prevent chemical cystitis, mesna was administered from day 1 to 4 during all cycles. Further supportive care included antiemetic agents, hyperhydration, methylene blue in patients with ifosfamide-induced encephalitis, and haematopoietic growth factors.

After the second cycle of VIP, new imaging was routinely performed, and based on the response assessment, a decision was made to either continue with 4 cycles of VIA or VIP or continue with the alternating schedule with 4 cycles of VIA-VIP until a favourable response was observed with both chemotherapeutic regimens. The criteria taken into account to distinguish between a good or bad response are described below (see Response Evaluation).

In the neoadjuvant setting (*n* = 34), 16 patients underwent local treatment after 4 cycles of chemotherapy, for example, after the sequence VIA-VIA and VIP-VIP. Local therapy consisted of radiotherapy, surgery alone, or surgery plus radiotherapy. The local treatment was based on the advice of a multidisciplinary tumor board. The preference for one or the other option was depending on the localization and size of the tumor, possibility for limb-sparing surgery, and the feasibility of surgery.

After local therapy, adjuvant chemotherapy for 4 cycles was administered (see Response Evaluation). In 18 patients, locoregional therapy was applied after completing the full 8 cycles of chemotherapy due to an insufficient response after 4 cycles. These patients received no further adjuvant chemotherapy.

Fifty-six percentage of patients completed the total of 8 cycles of chemotherapy, and on average, 7 cycles of chemotherapy were given. Most patients (14 patients out of 22) who stopped treatment prematurely had synchronous metastatic disease. In total, 29 patients (45%) needed a dose reduction, on average, after 3 cycles of chemotherapy. Main reasons for early therapy discontinuation or dose reduction were haematological intolerance or poor tolerance. Five patients (7.8%) received an upfront dose reduction either due to comorbidity or due to a cardiac disease localization.

### 3.3. Pathological Response in Patients Who Received Neoadjuvant Treatment

Among the 34 patients who received neoadjuvant treatment, 17 patients underwent surgery after 4 (*n* = 12) or 8 cycles of chemotherapy (*n* = 5). Sixteen patients underwent radiotherapy only. One patient died during the treatment before initiation of locoregional therapy.

Two patients with metastatic disease underwent surgery after induction chemotherapy: one patient underwent resection of lung metastases and the other patient had only very limited metastatic disease; therefore, based on a multidisciplinary board decision, the primary tumor was operated and the metastatic localizations were irradiated.

In total, we had a group of 19 patients undergoing surgery after chemotherapy with VIA/VIP. Five patients out of these (26%) had a pathological complete response (pCR). In another 6 patients (32%), more than 50% tumor necrosis was seen. In 8 out of 19 cases (42%), viable tumor tissue with necrosis less than 50% was seen.

### 3.4. Response Evaluation

**Figure 3:**
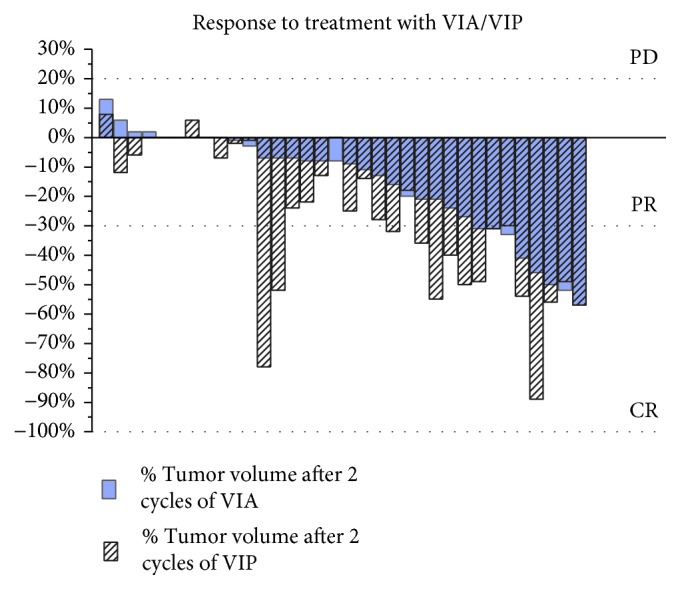
Radiologic response to chemotherapy with both VIA and VIP per patient (*n* = 34). Only evaluable patients are shown who had imaging after 2 cycles of VIA and 2 cycles of VIP. VIA: vincristine, ifosfamide, and doxorubicin; VIP: etoposide, ifosfamide, and cisplatin.

Among 64 ESFT patients treated with VIA/VIP, 7 (11%) were retreated with the same schedule due to recurrence of the disease. Because the typical dose of doxorubicin in the VIA/VIP schedule is 80 mg/m^2^, rechallenge with the same schedule is considered safe taking the cumulative doxorubicin dose into account. For patients diagnosed before 2000, no Digital Imaging and Communications in Medicine (DICOM) images were available (*n* = 11). Another 11 patients were treated in an adjuvant setting, as they had undergone a primary resection and had no postoperatively measurable disease according to RECIST. As a consequence, we were able to review the radiologic images of 42 patients, among whom 1 patient with recurrent disease undergoing rechallenge with VIA/VIP, generating 43 radiological response evaluations (Figure 3).

Among the 43 evaluations were 15 patients with metastatic disease, 27 patients undergoing neoadjuvant treatment, and 1 patient receiving additive VIA-VIP for residual disease after resection which could be followed radiologically.

For response assessment, CT-scan or MRI was used with equal frequency (53.5% versus 46.5%, resp.). In 32 out of 43 radiologically evaluable cases, more than one type of imaging was used to assess response: combination of CT and MRI in 17 cases (39.5%), combination of CT and nuclear imaging (positron emission tomography (PET) and bone scintigraphy) in 4 cases (9.3%), combination of MRI and nuclear imaging in three patients (7%), and combination of 3 or more imaging techniques in 8 patients (18.6%). Bone scintigraphy was used predominantly in patients with skeletal involvement while PET-CT was used in both neoadjuvant and metastatic settings independently of primary tumor localization.

**Figure 4:**
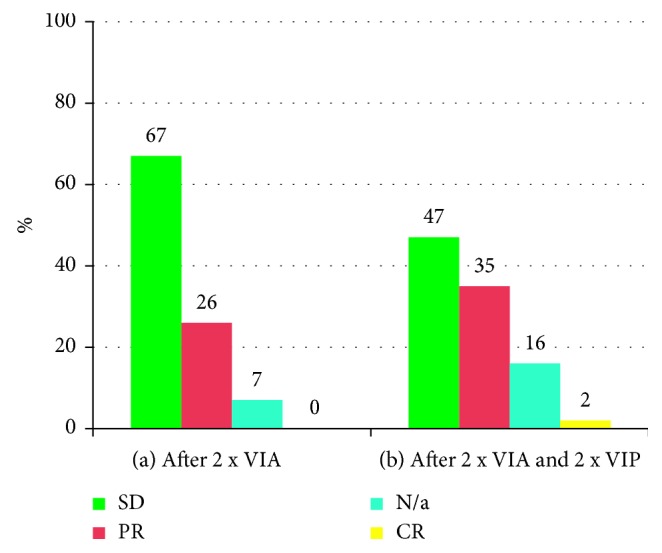
(a) Radiological response evaluation after 2 cycles of VIA and (b) radiological response evaluation after 2 cycles of VIA and 2 cycles of VIP according to RECIST 1.1. SD: stable disease; PR: partial response; CR: complete response; N/a: nonevaluable; RECIST: Response Evaluation Criteria in Solid Tumors; VIA: vincristine, ifosfamide, and doxorubicin; VIP: etoposide, ifosfamide, and cisplatin.

After the first 2 cycles of VIA, 11 out of 43 radiologically evaluable patients (26%) had a partial response (PR), 29 patients (67%) had stable disease (SD), none had progressive disease (PD), and 3 patients were not evaluable after the first 2 cycles because they had no imaging at this time point (Figure 4(a)).

Subsequent administration of 2 cycles of VIP led to a further reduction of the disease (PR after initial SD) in 5 patients (11%) and to confirmed SD in 30 patients (70%). Eight patients (19%) were not evaluable due to usage of other imaging techniques rather than baseline, or because they had no imaging at this time point.

The overall response rates after ending treatment with 2 cycles of VIA and 2 cycles of VIP was 37%, and the disease control rate was 84%: SD in 47%, PR in 35%, and complete response (CR) in 2%. Data were insufficient for evaluation in 16% of patients due to the reasons mentioned above (Figure 4(b)).

A total of 36 patients (84%) continued with chemotherapeutic treatment after the first 4 cycles. 28 out of 43 patients (65%) continued with the alternating regimen (i.e., 4 cycles of alternating VIA-VIP). Five patients (12%) continued with 4 cycles of VIA. Three patients (7%) continued with 4 cycles of VIP, the main reason being intolerance to doxorubicin (gastrointestinal intolerance and hepatotoxicity). There was one patient who responded only to the cisplatin-containing regimen. Seven patients (16%) received no further chemotherapy because of side effects.

### 3.5. Patient Outcome

One patient died prematurely after the first cycle of chemotherapy due to sepsis. There were no other chemotherapy-related deaths.

The median time of follow-up for all patients (*n* = 64) was 39.6 months. At the time of analysis, 32 out of 64 patients (50%) were alive.

All patients with metastatic disease had died (*n* = 19). The patient with the longest survival lived more than 7 years with metastatic disease at initial diagnosis. Median time between diagnosis and death was 13.3 months (5–88 months), and five-year overall survival was 5.3% in this patient group.

From the 45 patients with localized disease at the start of the treatment, 31 patients (69%) survived with a minimum follow-up period of 27 months. Five-year overall survival in this group of patients was 72.2%. One patient who developed metastases in lymph nodes 3 years after the initial diagnosis was retreated with the VIA/VIP regimen with complete response and received consolidation radiotherapy. He remained disease-free after 9 years of follow-up.

**Figure 5:**
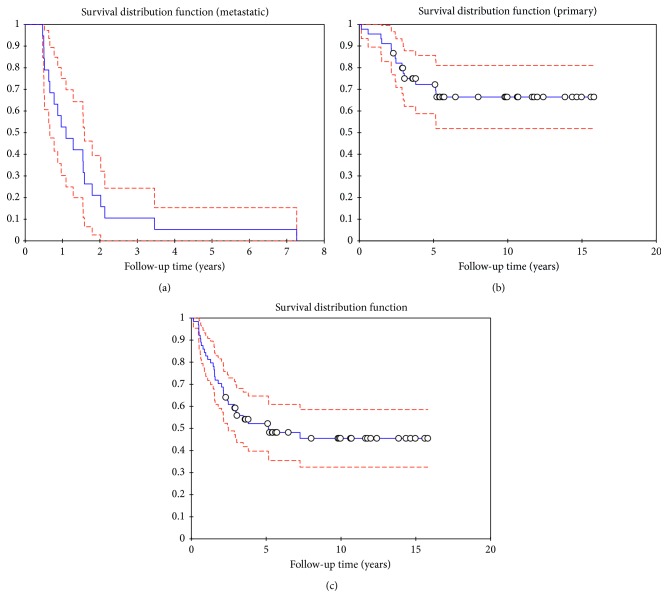
Kaplan–Meier curve survival. OS-curves for (a) patients with synchronous metastatic disease, (b) patients with primary ESFT without metastases, and (c) all patients (*n* = 64).

For the group of all patients, five-year overall survival was 52.2% (Figure 5).

## 4. Discussion

The use of alternating chemotherapeutic regimens is based on the Goldie–Coldman hypothesis that states that the proportion of resistant tumor cells increases over time and that alternating chemotherapy decreases the likelihood of mutations making tumor cells less resistant to a specific drug [22].

This rationale has led to several studies using alternating regimens in different types of cancers. In Burkitt lymphoma, the use of hyperCVAD (cyclophosphamide, vincristine, doxorubicin, and dexamethasone alternating with high-dose methotrexate and cytarabine) is now a standard treatment option [23].

Treatment with the alternating regimen VAC/IE (vincristine, doxorubicin, and cyclophosphamide alternating with ifosfamide and etoposide) is a standard approach in nonmetastatic ES [8]. Other alternating schedules are not routinely used in the treatment of nonmetastatic or disseminated ES. We have used the alternating triplet combinations VIA and VIP to allow sufficient dosage chemotherapy without obtaining the maximum tolerated dose.

Cisplatin has historically been little used in patients with ES, due to the fact that most of the patients are young, discouraging the use of this chemotherapeutic agent with possible severe late toxicity and morbidity such as renal insufficiency and hearing impairment.

Until today, there is no evidence that supports the use of cisplatin in the first-line treatment of ES. Carboplatin and cisplatin have however been used in recurrent or refractory ES [24–27]. The overall response rate (ORR) to ICE (ifosfamide, carboplatin, and etoposide) in children with recurrent ES was 48% [25]. In one retrospective European study conducted in 106 patients, carboplatin-etoposide was compared to cisplatin-etoposide in patients with refractory ES. Mean age was 20 years (range 2–48) in the carboplatin and 25 years (range 5–46) in the cisplatin group. Five-year overall survival was 24.5% in the carboplatin and 20% in the cisplatin group [26]. VIP as a second-line treatment has been evaluated in adult patients with refractory or recurrent ES in 27 patients [27]. A somewhat different dosing schedule was used compared to our centre (etoposide 75 mg/m^2^, ifosfamide 1200 mg/m^2^, and cisplatin 20 mg/m^2^ on days 1–5 in a three-weekly schedule). Median age was 18 years (range 16–34), and established ORR was 34%, which is good in this setting in an adult patient group.

The results of our analysis reveal that the overall survival for patients with localized disease is comparable to other reports using more common treatment options such as VAC/IE, VIDE, and VAIA, despite the fact that our patient group consisted of a relatively large number of patients with central bone disease (37.5%) which is known to have a negative impact on prognosis.

Cisplatin has given a favourable response to a subgroup of patients, as documented in Figure 3.

Chemotherapeutic adherence to the VIA/VIP schedule was lower in patients with metastatic disease due to worse tolerance, leading to premature discontinuation of the treatment and more dose reductions. This raises the question whether the VIA/VIP schedule might be too intensive in the metastatic setting.

About 50% of the patients were evaluated with MRI, using RECIST and assessing tumor size over time. RECIST could, however, not be applied in bone lesions. In evaluations with MRI, indirect signs of response to treatment such as tumor necrosis and oedema can be assessed. The actual activity of the regimen may therefore be underestimated by RECIST 1.1 response evaluation.

Noteworthy is the high percentage (26%) of pathological complete responses (pCR) in our patient group. Reported pCR rates are approximately 20% depending on the chemotherapeutic regimen that has been used [28]. The high rate of pCR in our series also supports the use of the VIA/VIP regimen in this setting.

## 5. Conclusion

Treatment of adult patients with ES remains challenging because of the aggressiveness of the disease and lack of standard treatment options.

We treated 64 patients aged 16 years and older with diagnosis of ES with the alternating chemotherapeutic schedule of VIA and VIP. ORR and OS were not inferior compared to reported results using multidrug regimens such as VAC/IE, VIDE, and EVAIA. Using an alternating regimen allows sufficient dosage of antitumor agents with a minimum risk of cumulating toxicity.

The use of cisplatin in first-line needs to be confirmed in a larger, randomized trial, but VIP has proved efficacy in recurrent or refractory disease. Taken into account possible late side effects of cisplatin, caution is warranted using this schedule in younger patients.

Given the fact that, in our analysis, more dose reductions and premature discontinuation of treatment were seen in the metastatic group and that outcome in this group remains very poor, we recommend careful patient selection.

## Figures and Tables

**Table 1:** Patient demographics.

Characteristic	No. of patients (total 64)	—	%
Age at diagnosis			
Median	—	26	—
Range	—	16–67	—
Sex			
M/F	38/26	—	59/41
Localization of primary tumor			
Bone, central	24	—	37.5
Bone, peripheral	14	—	21.9
Soft tissue, abdominal	9	—	14.1
Soft tissue, mediastinal	3	—	4.7
Soft tissue, subcutaneous	2	—	3.1
Soft tissue, other	5	—	7.8
Askin	6	—	9.4
Unknown	1	—	1.6
Metastatic disease at diagnosis	19	—	29.7
Adjuvant therapy	11	—	17.2
Neoadjuvant therapy	34	—	53.1
